# Structure and composition of grain boundaries and their impact on functional properties of energy materials

**DOI:** 10.1557/s43577-025-01038-y

**Published:** 2026-02-25

**Authors:** Oana Cojocaru-Mirédin, Elisa Wade, Yuan Yu, Jian Luo

**Affiliations:** 1 Department of Sustainable Systems Engineering (INATECH), University of Freiburg, 79110 Freiburg, Germany; 2 Institute of Physics (IA), RWTH Aachen University, 52056 Aachen, Germany; 3 Aiiso Yufeng Li Family Department of Chemical and Nano Engineering, University of California San Diego, La Jolla, USA; 4 Program in Materials Science and Engineering, University of California San Diego, La Jolla, USA

**Keywords:** Energy materials, Grain boundary, Grain-boundary functional properties, Atomic structure, Nanoscale characterization, Atom probe tomography

## Abstract

This article explores the impact of grain boundary structures and compositions on the functional properties of various materials for photovoltaics, batteries, and other energy-related applications. Examples of correlative microscopy studies highlight the potential to discover structure–property relationships at grain boundaries, essential for the design of energy devices to achieve superior performance. A grain boundary transition that promotes grain growth and reduces the boundary resistance in solid electrolytes is given as an example. A key focus will be on transport phenomena at grain boundaries, including mass, thermal, electrical, and ionic transport mechanisms. These transport phenomena are directly correlated with the charge defects that lead to a buildup of electric charges and potential barriers at the grain boundaries. In addition, applied electric fields can also induce boundary transitions that can affect grain boundary transport and other properties. Finally, we demonstrate that these potential barrier heights can be tuned by modulating the chemical composition, structure, and carrier concentration of the grain boundaries.

**Graphical abstract:**

**Figure:**
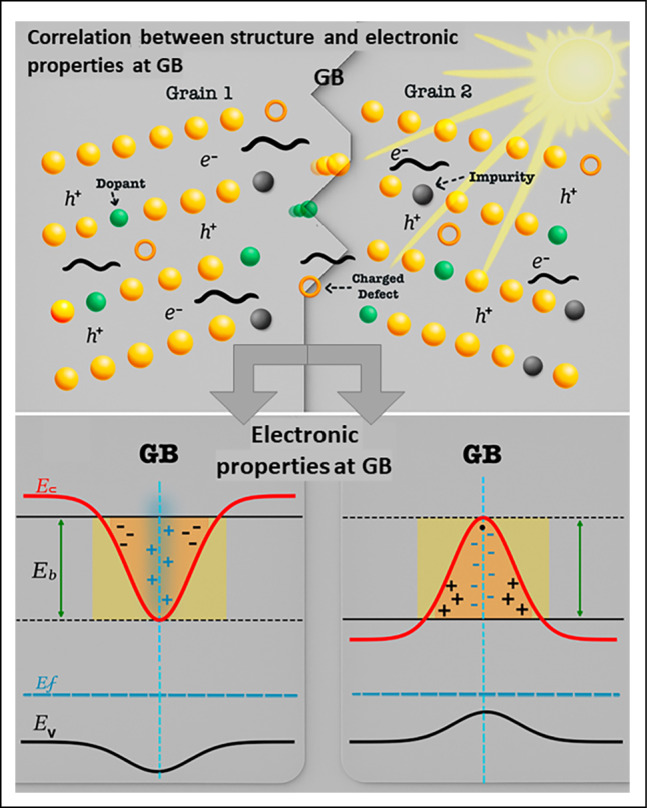
Obtaining grain boundaries (GBs) with superior properties based on the correlation between the structure, composition, and electronic properties at the GB level.

**Supplementary Information:**

The online version contains supplementary material available at 10.1557/s43577-025-01038-y.

## Introduction

Grain boundaries (GBs)—the intersection region between two neighboring grains of small crystals—have often been neglected in material design. This is in part because early studies considered that the material’s properties are closely related to grain interior (bulk) properties rather than GB properties, given that the bulk volume fraction is dominant. In addition, it is challenging to understand and quantify GB properties. In the early 1980s, the “GB engineering” concept was introduced, but with the focus on maximizing special GBs.^1^ Although this approach has been proven to be helpful in further improving the overall material performance, growing only special GBs, such as twin boundaries, has reached its limit in terms of the rational design of materials’ properties. To accurately assess these effects, the properties of GBs must be determined independently from those of the bulk. However, this remains challenging for energy materials because it is difficult to isolate and characterize the intrinsic properties of GBs separately from the bulk.^2–4^

Over the past decade, considerable efforts have been made to determine the properties of GBs in energy materials, including solar, battery, and thermoelectric materials. GB properties, including structure, composition, and transport, are of great interest for energy-harvesting semiconducting materials. GBs have very diversified structures, being classified as general and special, tilted and twisted, symmetric and asymmetric, as well as coherent and incoherent. Moreover, their distinct types based on disorientation angle (three main categories: symmetric Σ boundaries, low-angle GBs (LAGBs) with a disorientation angle below 15°, and random high-angle GBs (RHAGBs) with a disorientation angle above 15°), GB plane, curvature, and atomic structure are crucial as well.^5^ The determination of their distinct functional properties, such as transport properties, is even more challenging, which explains why this has remained elusive until now. A GB creates a potential fluctuation in the periodic atomic potential, resulting in a potential barrier that may affect the transport of electrons, holes, phonons, and ions across or along the interface and impact properties such as electrical, thermal, and ionic conductivity.^6–8^ The magnitude of this barrier depends on many factors such as the GB’s structure, chemistry, and chemical bonding.^9–11^ Moreover, the GB structure and chemistry also lead to a local change in the density of states and charge density when compared with the grain interior (or bulk).

Although challenging, several experimental and computational methods have been developed and employed for the GB characterization. Attempts in determining structure–chemistry relationships at GBs were realized by employing correlative microscopy approaches such as atom probe tomography (APT) correlated with electron backscatter diffraction (EBSD) or scanning transmission electron microscopy (STEM).^12–18^ Some of these studies have demonstrated that there is indeed a 1:1 correlation between composition and structure, not only at the micrometer level, but especially at the atomic level,^15^ proving that the determination of the GBs’ atomic structure is critical. While energy materials encompass a wide range of properties (such as structure and composition), the electronic properties play a dominant role in the function and performance of energy devices. Therefore, there is a growing need to understand not only the GBs’ electronic properties in energy materials,^19–22^ but also to relate these electronic properties to GBs’ structure and composition through correlative microscopy and techniques of experiments^14,23–27^ and simulations.^28,29^ Thus, in this article, we are summarizing the existing work on GB properties in energy materials and will provide future directions necessary to be assessed in science concerning GB rational design.

## Atomic structure, electron transport, and charge defects at GBs in solar cell absorber materials

Photovoltaic solar cells are capable of transforming sunlight into clean and sustainable electrical energy. Traditionally, these cells are based on silicon (Si) wafers as an absorber, either amorphous or crystalline.^30^ It is also possible to produce solar cells based on thin-film absorbers, either from Si or other materials such as copper indium gallium diselenide (Cu(In,Ga)Se_2_, CIGS), cadmium telluride (CdTe), or perovskites.^30,31^ Compared to traditional Si, thin-film technology requires thinner layers, which is more cost-effective due to less material use, and can be utilized to make solar cells lighter and more flexible for better integration.^30,32^ Thin-film solar cells can achieve efficiencies close to or exceeding traditional Si cells, especially with tandem designs.^33,34^

GBs have a particularly strong impact on thin-film solar cells compared to traditional (bulk or single-crystal) solar cells, because thin films are often polycrystalline with small grain sizes and high GB density. These boundaries can act as sites for charge recombination, ion migration, and can significantly affect the efficiency of the device.^35,36^ GBs in thin films are dominant sites for nonradiative recombination, where charge carriers (electrons and holes) recombine without generating electricity, reducing efficiency.^36,37^ They can further serve as channels for ion migration, which can lead to device instability and faster degradation, especially in perovskite and halide-based thin films.^36,37^ Smaller grains, which mean more boundaries, in thin films often lead to worse performance; increasing grain size or passivating boundaries can improve efficiency.^35,38,39^

**Figure 1:**
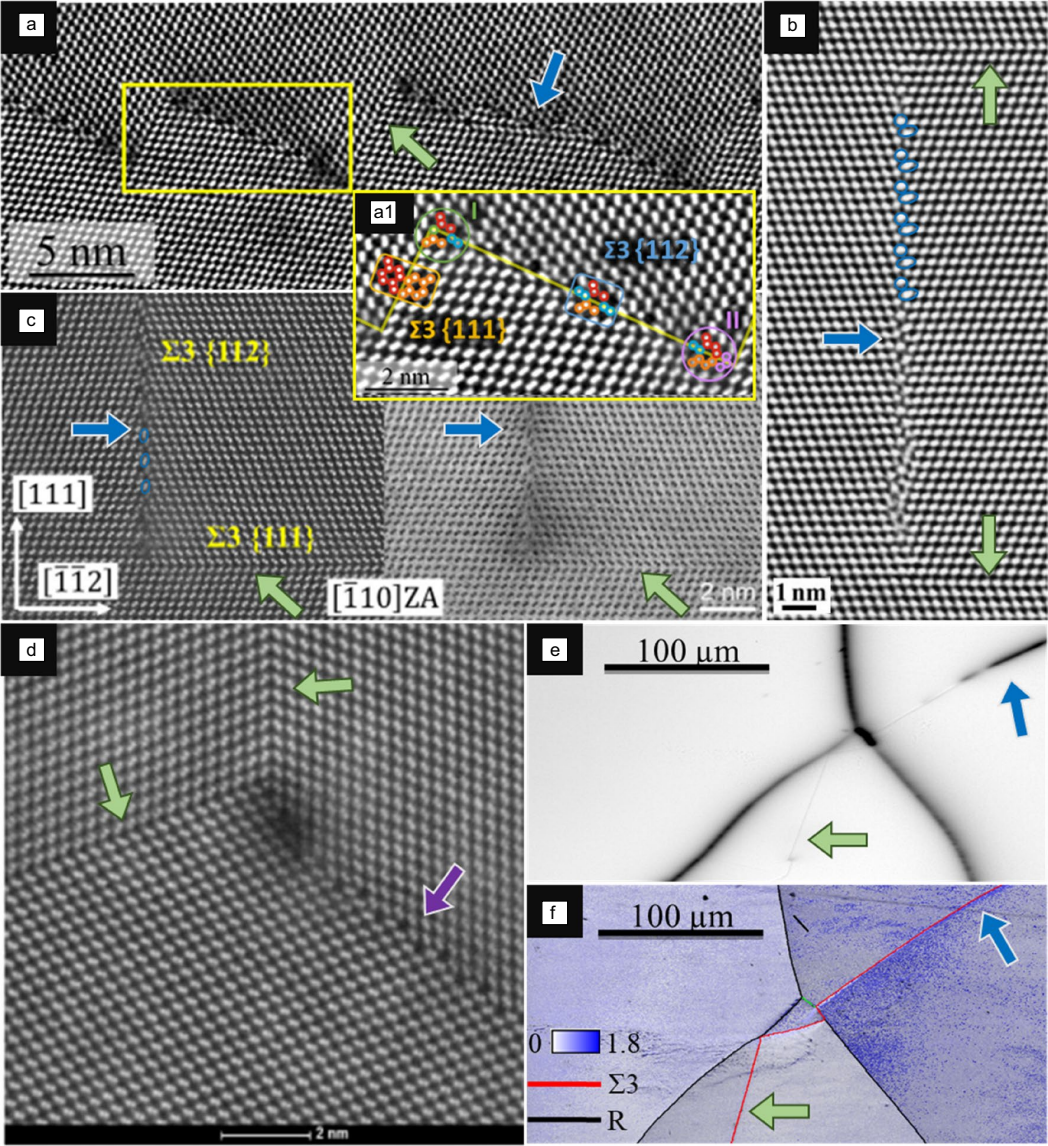
Atomic structures of several Σ3 grain boundaries (GBs) in silicon. The {111} Σ3 GBs are indicated with light green arrows, while {112} Σ3 GBs are shown using a blue arrow. In (b) and (c), a few atoms not clearly attributable to either grain are marked blue. (a) High-angle annular dark-field scanning transmission electron microscopy (HAADF-STEM) image resolving the faceted structure of a Σ3 GB in multicrystalline Si, the faceted GB consists of {111} Σ3 and {112} Σ3 components.^40^ The section highlighted in yellow is shown in more detail in inset a1.^40^ (b) A high-resolution image of a {112} Σ3 GB in polycrystalline Si, with two {111] Σ3 GBs to the right of it.^41^ (c) HAADF-STEM (left side) and bright-field-STEM (right side) image of a GB junction of two Σ3 GBs in multicrystalline Si.^42^ (d) High-resolution HAADF-STEM image of a triple junction in multicrystalline Si. The three GBs meeting at the junction have a symmetric structure, the upper and left Σ3, and right Σ9 (indicated by the purple arrow).^43^ (e) Electron-beam-induced current map of an area of multicrystalline Si, showing multiple GBs. (f) Scanning electron microscopy image of the sample area in (e) overlaid with electron backscatter diffraction information on GB crystallography. The deviation in orientation from 0° to 1.8° inside the grains is plotted in the map. (b, d, e, f) Adapted with permission from References 41, © 2007, Springer Nature, and 43, 2017, RWTH Aachen University. (a) Preprinted from Reference 40. © 2018 American Physical Society. (c) Reprinted from Reference 42. © 2020, AIP Publishing.

The atomic structure of GBs is not identical to the surrounding grains by their very nature. Where the different grains of the material intersect, an intermediate structure results. These intermediate structures at the GBs can be very orderly, often visible at coherent, symmetric GBs. **Figure ****1** shows a selection of such Σ3 GBs (also called twin boundaries) in multicrystalline or polycrystalline Si GBs investigated using high-angle annular dark-field scanning transmission electron microscopy (HAADF-STEM) and bright-field (BF)-STEM.^40–44^ The atomic structure of all of these GBs is periodic, with a small intermediate region.

The {111} Σ3 GBs and facets indicated by green arrows in Figure 1a–d^40–44^ show a minimal intermediate region, with each atom of one grain aligned with one of the other, effectively mirroring it, with minimal disruption of the structure, and all atoms can be attributed to either grain. For the {112} Σ3 GBs and facets indicated by blue arrows in Figure 1a–c,^40–42^ the intermediate region is more substantial, with some atoms that cannot be clearly attributed to one of the two intersecting grains interspersed. A few such examples are marked in blue in Figure 1b–c, as well as by Liebscher et al.^40^ in Figure 1a. There is a pattern visible of the atoms’ arrangement within the GB repeating periodically in all three {112} Σ3 GBs.

Figure 1e–f shows the same area of multicrystalline Si with multiple intersecting GBs. Figure 1e contains an electron-beam-induced current (EBIC) map of the area, which shows some of the GBs to exhibit no recombination activity (0% contrast for {111} Σ3 GBs, shown by green arrow in Figure 1e), medium (20–30% contrast for {112} Σ3 GBs, shown by blue arrow in Figure 1e), or very high recombination activity (random HAGBs and triple points).

It was proven indeed that the recombination activity increases by decreasing the symmetry and coherence of GBs because impurities are more prone to be accumulated there.^45^ This is because by decreasing the symmetry and coherence of the GB, the GB structure becomes larger and more disordered and defective, allowing impurities to accumulate. This is visible in higher asymmetry grain boundaries as well, such as Σ9 GBs presented in Figure S1 and Figure 1d. This intermediate region could be interpreted as a separate phase, previously found to be the case for Cu by Meiners et al.,^46^ and possibly the same for the Si regarded here. If the intermediate phase is large, it follows that the change in the materials’ properties at this location may also be large, making GBs a potential target for material modifications to make these properties beneficial. Moreover, the faceted GB in Figure 1a contains short Σ3 {111} and long Σ3 {112} facets with different atomic structures for different inclinations (different GB phases), indicating a GB faceting transition. Hence, this could represent an example of interfacial phase transformation that takes place at the GB region.

Not only the atomic structure, but also the chemical composition and electrical properties of these boundaries can either enhance or hinder solar-cell performance, depending on their specific traits. The influence of GB composition is notable: certain elemental distributions and charge defect types at GBs can be beneficial, whereas others are detrimental to device efficiency.^47,48^

Where the elemental composition of GBs in Si is not a factor, the chemical elemental composition of GBs in CIGS thin films contributes significantly to determining their electrical properties and, consequently, the performance of CIGS solar cells. Variations in elemental composition at GBs can make them beneficial, neutral, or detrimental to device efficiency, with Cu depletion at GBs generally linked to improved performance.^39,48,49^ The solar cells benefit from certain compositional and structural features, such as Cu depletion, increased Se, and the presence of alkali dopants.^39,48^ However, detrimental factors include Cu enrichment, Se and Ga depletion, as well as a low Na content.^39,48^ Careful adjustment of GB chemistry can enhance device efficiency by reducing recombination and optimizing electrical properties.^48,49^

In the case of CdTe solar cells, the functioning cells are often treated with Cl, and the segregation of Cl to GBs is generally considered beneficial,^50^ due to the GBs that are passivated through this Cl doping. This passivation occurs as Cl atoms substitute for Te at the GBs, creating local *p*–*n*–*p* junctions.^51^ These junctions can enhance carrier collection and reduce recombination, making this process generally more beneficial for the overall performance of CdTe solar cells.^51,52^

CIGS solar cells can benefit from alkali passivation.^48^ Alkali doping (Na, K, Cs) at GBs passivates charged donor defects, reducing energy barriers for holes and increasing both hole mobility and free carrier concentration.^47^ Doping decreases the activation energy of conductivity by lowering the GB barrier height, facilitating better charge transport across grains.^47,53,54^ This leads to improved conductivity and reduced recombination losses at GBs.^53^

The electrical nature of GBs can determine whether they are benign or detrimental to the cells’ effectiveness, but in thin films, detrimental effects are more common due to the presence of higher recombination-active charge-defect densities.^38,47^ In polycrystalline and multicrystalline Si thin films, charge defects (i.e., point defects which trap charge carriers^55^) are found both at GBs and within grains. For larger grains, intra-grain defects become the dominant performance-limiting factor, while in smaller grains, GB defects are more significant.^56^

In CIGS solar cells, donor defects have a negative impact, while acceptor defects can be beneficial or neutral.^38^ The presence of surface donors at GBs creates hole barriers, which can affect hole concentration and recombination rates. The impact of these barriers varies depending on the grain size and the composition of the GBs.^57^ Alkali atoms can localize both at GBs and within grains, passivating charge defects throughout the material. However, excessive doping can introduce new defects and degrade performance.^47,58^ In perovskite solar cells, a multitude of defect clusters were detected at the GB (containing a high density of interstitial iodine) and were proven to be highly detrimental, although some types of defects, such as lead iodide, are relatively benign.^59,60^ Across CdTe, Si, and CIGS solar cells, high defect densities lead to deviations from ideal device behavior, affecting efficiency and current–voltage characteristics.^60,61^ In Si solar cells, GBs act as recombination sites and serve as segregation sites for impurities such as carbon, nitrogen, and oxygen. O and C have both been shown to increase recombination activity in Si GBs, making them detrimental factors.^45,62^ Additionally, GBs in Si form charged vacancies, which form deep defect electronic states within the bandgap, making them generally more detrimental to solar-cell performance.^62^ Oxygen is a typical impurity in CdTe, possibly introduced during various steps of the fabrication process.^50^ While it is suspected to reduce the grain size of CdTe by acting as a nucleation aid and increasing downward band bending, its effects are difficult to isolate from other influences.^50^ There is little evidence of O at typical GBs, so it is not generally considered detrimental.^50,51^ In contrast, O has been found to be segregated at detrimental GBs in CIGS absorbers, while not found at benign GBs, making it a possible detrimental factor in CIGS.^48^

Although in some metallic systems, the influence of GB structure on the electrical properties is well known,^63^ in Si there is no such definitive link to date.^64^ Instead, a connection between the thermal resistivity and the CSL structure of GBs has been made by Isotta et al.,^65^ where LAGBs present with significantly lower thermal resistivity values than HAGBs. For CIGS solar cells, this link has not been established either. Instead, its electrical behavior is most closely tied to the local chemical composition, such as the In/Ga ratio.^66^

**Figure 2:**
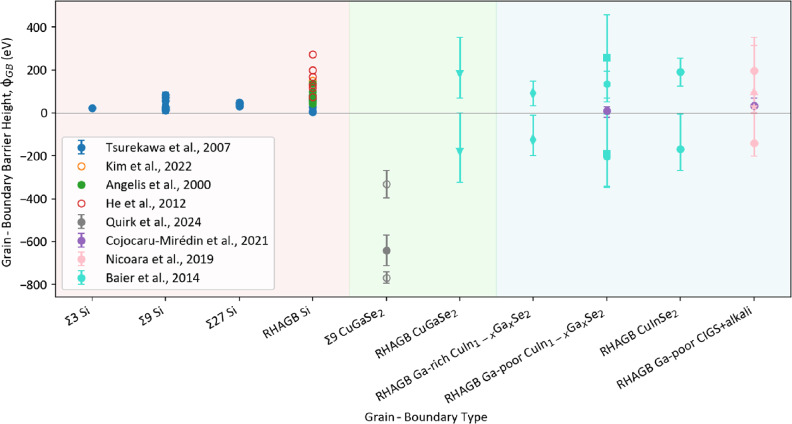
Grain-boundary barrier height *ϕ*_*GB*_ of Si and CIGS, dependent on their Σ-type and composition. The data included are taken from Tsurekawa et al.^68^ (blue), Kim et al.^69^ (yellow), Angelis et al.^70^ (green), and He et al.^71^ (red) for Si. The CIGS data are extracted from Quirk et al.^72^ (gray), Cojocaru-Mirédin et al.^39^ (purple), Nicoara et al.^47^ (pink), and Baier et al.^73^ (cyan). Simulated data are included using empty circles. Different shapes of the data points indicate different *x* = Ga/(In + Ga) ratios for the data by Baier et al.^73^ with *x* = 0.33 as squares, *x* = 0.45 as hexagons, and *x* = 0.75 as diamonds. For the data by Nicoara et al.^47^ different data point shapes indicate the different alkali dopants utilized (KF as circles, CsF as triangles, RbF as stars).

The barrier height of a GB in a material can provide an idea about the material’s electrical properties, such as the electrical resistivity and conductivity, as the GB acts as a potential barrier for the charge carriers’ transport inside the solar cell.^67^ A comparison of those barrier heights for Si and CIGS is shown in **Figure ****2**, where they depend on their Σ-type and, in the case of CIGS, their Ga/(In + Ga) ratio. Generally, the barrier heights of Si are positive, while those of CIGS can be either positive or negative. The barrier heights of Si^68–71^ ranging between 0 and approximately +300 meV are lower than those of CIGS,^39,47,72,73^ which range between 0 and approximately 450 mV and 0 and approximately –800 meV, indicating a lower resistivity of the Si GBs. Nonrandom GBs in Si show lower barrier heights than RHAGBs, while the nonrandom GBs measured for CIGS by Quirk et al.^72^ show a considerably higher absolute values than the RHAGBs measured in CIGS. Some alkali-dopants, such as NaF, CsF, and RbF show lower barrier heights than others (KF) and seem to reduce it as opposed to undoped CIGS (the alkali samples, both by Nicoara et al.^47^ and Cojocaru-Mirédin et al.^39^ have a Ga/(In + Ga) ratio of approximately *x* = 0.33).

In the case of CdTe solar cells, as previously mentioned, the electrical properties of GBs can be influenced by passivation with Cl, which creates local *p*–*n*–*p* junctions, enhancing carrier collection and reducing recombination, making this process generally beneficial. Certain types of GBs, such as coherent twin boundaries in CdTe, are less detrimental and can even enhance current transport due to a lower electron potential at the boundary compared to the grain interior.^51,52,74,75^ For Si and CIGS solar cells, passivation of GBs using H or small polar molecules can neutralize localized charges, reduce recombination, and enhance charge transport across boundaries.^76,77^

GBs and their defects are a significant factor in determining the performance of thin-film solar cells due to their high density and influence on charge recombination and electrical conductivity. Although their impact is often detrimental, appropriate engineering through structural control, doping, and chemical passivation may mitigate negative effects and possibly enhance device performance. These factors are not independent of each other. Understanding and tailoring GB properties and their interplay in solar cell materials like Si, CIGS, and CdTe is therefore beneficial for optimizing thin-film photovoltaic efficiency and stability.

## Effect of GBs on thermoelectric properties

The GB microstructures can also significantly influence the heat-to-electricity energy-conversion efficiency, as determined by the dimensionless thermoelectric figure of merit, zT = *S*^2^σ*T/*κ.^78^ Here, *S*, σ, κ, and *T* represent the Seebeck coefficient, the electrical conductivity, the thermal conductivity, and the absolute temperature, respectively. The optimization of zT depends on the meticulous manipulation of the interweaving transport of electrons and phonons.

The presence of GBs, in general, impedes the transport of electrons and phonons, reducing both electrical conductivity and thermal conductivity. Thus, the enhancement of zT by engineering GBs is only feasible if the phonons can be scattered more strongly than electrons at GBs. Indeed, the nanostructuring strategy has successfully enhanced the zT value of compounds such as Bi_0.5_Sb_1.5_Te_3_^79^ and PbTe^80^ due to reduced thermal conductivity. However, abnormally increased thermal conductivity has also been observed in polycrystalline SnSe compared to single-crystal SnSe.^81^ Regarding electron transport, the increased GB fraction can lead to significant electron scattering and, thus, even decrease the zT value. This phenomenon is prominent in materials such as Mg_3_Sb_2_,^82^ oxides,^83^ and half-Heusler alloys.^84^ For example, the room-temperature electrical conductivity and zT value of Mg_3_Sb_2_-based compounds can be increased by a factor of about five upon increasing the average grain size from 1 μm to 7.8 μm.^85^ Other examples show that the electrical conductivity can also be increased by tuning the GB composition while maintaining the grain size.^86–88^ These controversial effects of GB on the thermoelectric performance (improvement of zT) via engineering GBs remain elusive. Decoding the structure–property relationship for electron and phonon transport at GBs is a crucial step in designing thermoelectrics via tailoring GBs and microstructures.

**Figure 3:**
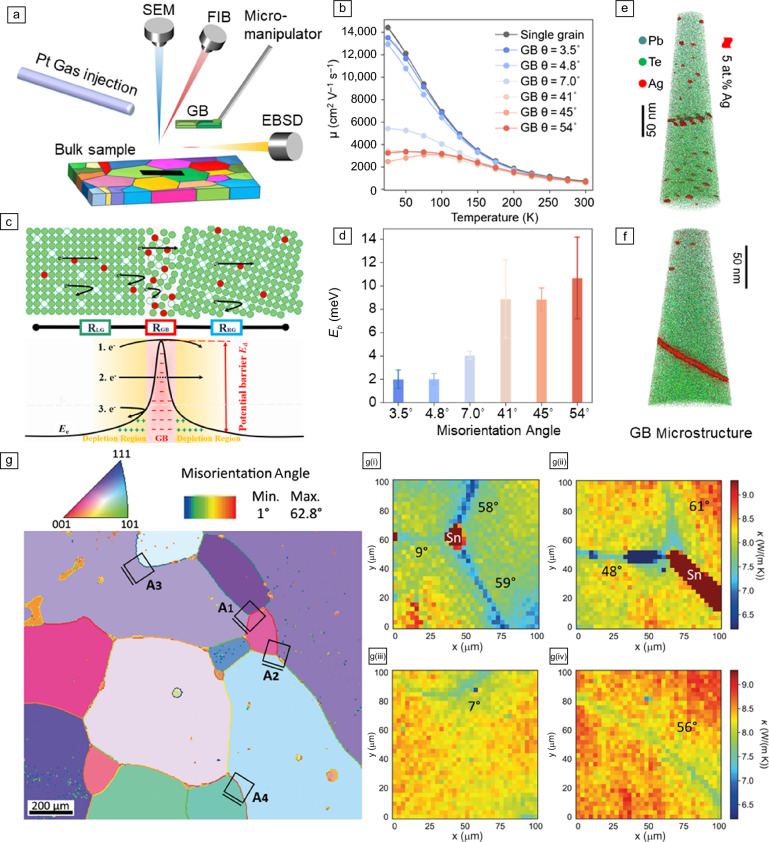
Effect of grain boundary (GB) on the electrical and thermal transport properties. (a) Schematics demonstrating the “liftout” of a lamella specimen, including one specific GB characterized by electron backscatter diffraction (EBSD). FIB, focused ion beam. (b) Temperature-dependent Hall carrier mobility of the lamellae specimens with various individual GBs. (c) Sketches illustrating the two-phase series-circuit model and the formation of GB potential barrier height due to spatial charges. (d) The GB potential barrier height for GBs with different misorientation angles derived from the data in (b). (e) Atom probe tomography (APT) reconstruction showing the Ag-decorated dislocation arrays at a low-angle GB of Ag-doped PbTe. (f) APT reconstruction showing the coverage of the whole high-angle GB plane by Ag. (g) EBSD inverse pole figure of a polycrystalline SnTe sample, showing the distribution of different GBs. The squares A1 to A4 indicate the regions of interest for frequency domain thermoreflectance measurements. (g[i]–g[iv]) show the spatially resolved thermal conductivity corresponding to regions A1 to A4 indicated in (g). Adapted from References 26, © 2023, Springer Nature, 90, © 2024, American Chemical Society, and 97, © 2023, Wiley.

It should be emphasized that the previously discussed examples only demonstrate the average effect of GBs on thermoelectric properties, which do not reveal the one-to-one correlation between individual GB microstructures and electron/phonon transport. Some emerging studies utilizing correlative characterization methods bring a new opportunity to address this dilemma. **Figure ****3**a illustrates the preparation of a microscale lamella specimen, including one individual GB, using an SEM–FIB dual-beam system attached with EBSD. Specific GBs will be first screened by EBSD and then lifted out by a focused ion beam (FIB) assisted with a micromanipulator and Pt gas injection system. The lamella specimen will finally be transferred onto a predeposited microcircuit and loaded into the physical property measurement system (PPMS) for transport property measurements. More details about the whole process can be found in these articles.^26,89^ By utilizing this protocol, temperature-dependent electrical conductivity and carrier mobility for various lamella samples with different GB misorientation angles can be determined. Figure 3b shows that the carrier mobility decreases negligibly when electrons pass through low-angle GBs compared with the GB-free sample of Ag-doped PbTe. In stark contrast, the mobility is significantly reduced in samples with high-angle GBs, especially at low temperatures when the GB electron scattering is more prominent. This implies that the presence of high-angle GBs strongly impedes the transport of charge carriers. Figure 3c sketches a two-phase series-circuit model with the GB area described as a new phase different from its adjacent grains.^22,90^ The increased GB resistance can be underpinned by the establishment of a potential barrier induced by the spatial charge area near GBs. For the GB-dominated charge transport process, the carrier mobility can be expressed by:^91^

1 $${\upmu }_{\mathrm{GB}}=Le{ \left(\frac{1}{2\uppi {m}^{*}{k}_{\mathrm{B}}T} \right)}^{\frac{1}{2}}\mathrm{exp} \left(\frac{-{E}_{\mathrm{b}}}{{k}_{\mathrm{B}}T} \right),$$

where *L* is the grain size, *e* is the electron charge, m^*^ is the effective mass of charge carriers, *k*_B_ is the Boltzmann constant, *T* is the temperature, and *E*_b_ is the potential barrier height. Thus, the *E*_b_ can be derived from the temperature dependence of the GB mobility. Figure 3d shows that the *E*_b_ value for low-angle GBs is about five times lower than that of HAGBs. This potential barrier height depends on the number of trapping states and the static dielectric permittivity at the GB. Figure 3e–f shows the 3D-APT reconstruction of a LAGB sample and a HAGB sample used for PPMS measurements, respectively. While Ag only segregates to the dislocation arrays at the LAGB, these impurity atoms can cover the whole HAGB plane. Quantitative Gibbs excess of Ag atoms at the GB indicates that the number of trapping states at the HAGB is about 1.5 times higher than that at the LAGB. This is partly responsible for the larger *E*_b_ value of HAGBs. Another factor that leads to the larger *E*_b_ is the collapse of metavalent bonding at HAGBs, which can also be unraveled by APT measurements.^26,90^ Interested readers are encouraged to refer to these articles about the relationship between chemical bonding and thermoelectric properties,^92–94^ as well as the powerful capability of APT in probing local bonds.^95,96^ The previously discussed correlative characterization methods can be easily extended to other materials such as PbSe^90^ and PbS,^89^ to explore more uncharted territory of how GBs affect transport properties.

As thermoelectric performance is determined by both the electron and phonon behavior, it would be ideal to also correlate the phonon properties across individual GBs akin to the electrical properties previously introduced. Isotta et al.^97^ utilized a frequency-domain thermoreflectance (FDTR) method to map the spatially resolved thermal conductivity of SnTe, in conjunction with corresponding GB features characterized by EBSD. Figure 3g shows an inverse pole figure of binary SnTe with four typical GB areas indicated as A1–A4. Their spatial distribution of thermal conductivity is shown in Figures g(i–iv), respectively. GBs with large misorientation angles show higher thermal conductivity than LAGBs, which is akin to the carrier mobility behavior across GBs. These results imply that HAGBs scatter both electrons and phonons more prominently than LAGBs. Some open questions remain for this FDTR technique because only the total thermal conductivity can be measured. It is hard to distinguish the contribution of electron and phonon scattering at GBs to the suppressed total thermal conductivity. The combination of FDTR with the FIB-PPMS-based methods could be a complementary and promising strategy to fully uncover the effect of individual GBs on thermoelectric properties. Structural characterizations such as STEM and APT from the same GB area offer invaluable features to explain these transport properties. As a consequence, we can realize the ultimate goal of designing high-performance thermoelectrics by tailor-made individual GBs.

**Figure 4:**
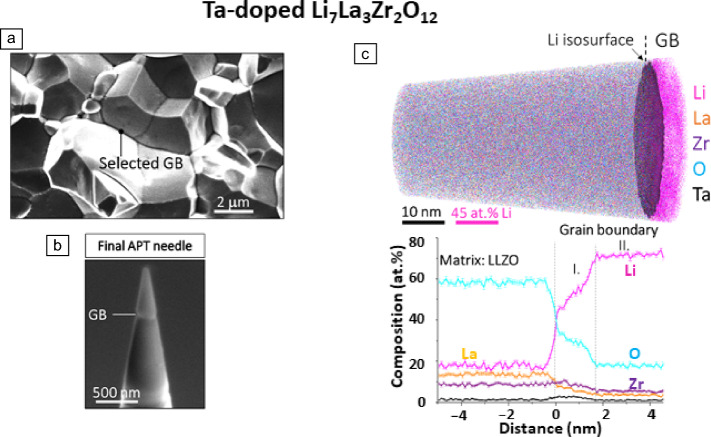
Atom probe tomography (APT) investigation of the solid ceramic electrolytes for Li-ion and Na-ion batteries. ^18,111^ (a) Scanning electron microscopy image highlighting the grain boundary (GB) selected for Ta-doped Li_7_La_3_Zr_2_O_12_ for the APT study. (b) APT needle containing the GB selected. (c) Three-dimensional APT map showing the distribution of Li (pink), La (orange), Zr (violet), O (light-blue), and Ta (black) atoms. The GB position is highlighted by a Li isosurface constructed by employing an iso-composition value of 40 at.% Li. The proximity histogram below has been constructed using the given Li isosurface. The “0” position marks the interface between the grain (left) and the GB (GB, right). Reprinted with permission from Reference 18.

## Effect of GBs on mass and ionic transport in battery materials

### GB stoichiometry for battery materials

Many attempts have been made in the past to determine the exact stoichiometry and structure of GBs down to the atomic level in battery materials. This is due mainly to the substantial challenge in the direct imaging and quantification of light alkalis, such as Li or Na atoms, by many analytical techniques. Although x-ray spectroscopy (EDXS) in electron microscopy could prove qualitatively the existence of an approximately 1-nm-thick glassy phase at the GBs of the Li_1–2*x*_Ca_*x*_Si_2_N_3_ ceramic^98^ or the infusion of LPO solid-state electrolyte along the GBs of secondary particles in a Ni-rich NMC layered cathode,^99^ the GBs’ exact atomic structure and stoichiometry remain inaccessible. This is because imaging a region-of-interest of the battery materials, which are beam-sensitive materials, with a long probing time and/or large current can seriously damage this region, already after the first acquisition (so-called electron-beam irradiation). Yet, it has been proven that the usage of cryo-electron microscopy^100,101^ or imaging conditions under very low acquisition times^102^ allows a qualitative identification of Li redistribution as well as an identification of the battery material structure with nearly atomic resolution (without beam damage), but not yet at the GB level. This gap in knowledge hinders full-atomic-scale characterization of the battery materials, given that knowledge on the alkali-induced interphase formation, alkali segregation, and/or alkali conductivity (diffusion) is known to be of paramount importance to understand the link between composition, structure, and electronic properties and, hence, to understand the mechanism responsible for the improvement of the battery cell performance.

In contrast, besides its 3D capabilities and near-atomic resolution, the APT detects both light and heavy elements, with equal probability, as shown for various energy materials containing Li, Na, or Rb.^12,103–110^ Recently, APT studies were done on the GBs of the ceramic electrolytes,^18,111,112^ some of which are summarized in **Figure ****4**. All these studies show that the width of the GB (5 nm or greater) is much greater than the typical structural width of 0.5 nm and that alkali ions are strongly accumulated at the GB region, as shown in Figure 4 with values of about 70 at.% for Li and 40 at.% for Na (not shown here). This scenario is very different than the typical segregation phenomenon observed, for example, in the case of Sm-doped ceria, where 0.4 at.% Sm is segregating at the 1.6-nm-thick GB.^113^ In the case of Li_7_La_3_Zr_2_O_12_, Na_3.4_Zr_1.4_Si_2.6_P_0.9_O_11.95_, and Li_(1+*x*)_Al_(*x*)_Ti_(2−*x*)_(PO_4_)_3_ ceramic electrolytes, two-dimensional (2D) interfacial phases are formed at the GBs.^18,111,112^

### Interfacial phases in solid electrolytes and battery materials

**Figure 5:**
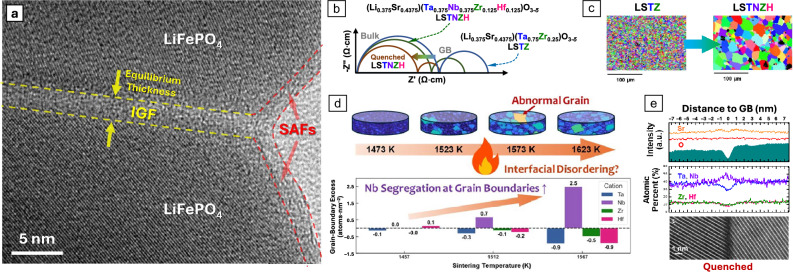
Grain boundary (GB) defect phases in solid electrolytes and their direct and indirect roles in promoting ionic conductivity. (a) Analogous phosphate-based 2D interfacial phases (intergranular films [IGFs] and surface amorphous films [SAFs]) formed at GBs and free surfaces in LiFePO_4_, providing a fast Li^+^ transport pathway^122^ (b–e). An example of utilizing a GB transition to improve the conductivity of solid electrolytes. (b) In a new class of compositionally complex perovskite oxides, improved lithium ionic conductivities are attributed to a GB transition that enhances grain growth and improves specific GB conductivity.^132^ The ionic conductivity is improved by >60% in (Li_0.375_Sr_0.4375_)(Ta_0.375_Nb_0.375_Zr_0.125_Hf_0.125_)O_3-δ_ (LSTNZH), compared to the (Li_0.375_Sr_0.4375_)(Ta_0.75_Zr_0.25_)O_3-δ_ (LSTZ) baseline. Furthermore, the ionic conductivity is improved by another >70% via quenching (to preserve more disordered GBs with Nb segregation), achieving >270% of the LSTZ. (c) The enhanced grain growth in LSTNZH reduces the total GB resistance. (d) Notably, increasing temperature induces significant GB segregation of Nb, in contrast to the classical GB segregation models that predict temperature-induced desorption but suggest premelting-like GB disordering coupled with Nb_2_O_5_ segregation, which resulted in abnormal grain growth.^133^ (e) Quenched specimens show more disordered GBs with Nb segregation that can also lead to increased specific GB conductivity^132^ (a) Adapted with permission from Reference 122. © 2009 AIP Publishing. (b), (c), and (e) Adapted from Reference 132. © 2023 CC‐BY license. (d) Adapted from Reference 133. © 2025 CC‐BY license.

Interfacial phases, also known as “complexions”^114^ or interfacial defect phases that are thermodynamically 2D,^115^ can be utilized to tailor solid electrolytes and battery materials, which have been discussed in two prior review articles.^116,117^ On the one hand, the formation of silicate-based intergranular films (IGFs) that adopt an equilibrium thickness,^118,119^ a common type of GB defect phase widely observed in ceramics, can often be detrimental for ionic conduction. For example, silicate-based IGFs can form and block the oxygen-ion conduction in Y_2_O_3_-stabilized ZrO_2_ (YSZ) based fluorite oxides.^120^ and proton conduction in perovskite oxides.^121^ On the other hand, phosphate-based, amorphous-like 2D interfacial phases can form spontaneously at both free surfaces and GBs in annealed battery cathode particles to provide a fast Li^+^ conduction pathway, as exemplified in the case shown in **Figure ****5**a.^122^ In addition, the formation of nanoscale glass-like IGFs in lanthanum phosphate solid electrolytes was found to increase proton conductivity.^123^ Moreover, the oxygen-ion conductivity of La_2_Mo_2_O_9_ nanowires can be increased substantially^124^ via fast conduction in nanoscale surface amorphous films (SAFs),^125^ a surface defect phase that can be considered as the free surface counterparts^125^ to the widely observed IGFs^118^ in ceramics. Notably, phosphate-based SAFs with a nanoscale self-limiting or “equilibrium” thickness (Figure 5a) have been utilized to improve the charge capabilities and cycling stability of lithium-ion batteries.^117,122,126–129^

These IGFs and SAFs can alternatively be considered as disordered multilayer adsorbates (albeit the existence of partial orders) formed at thermodynamic equilibria.^118,125,130^ We note that adsorbate-based 2D interfacial phases at GBs can adopt 2D crystal-like, amorphous-like, or even quasicrystal-like structures that are distinct from bulk phases.^131^ In addition to IGFs and SAFs previously discussed, other types of defect phases can also form and affect the performance of solid electrolytes and batteries.

Furthermore, a first-order or continuous GB phase-like (complexion) transition can be induced by changing temperature or chemical potential.^114,131^ Here, GB phase diagrams can be computed to represent the stability and transitions of 2D interfacial phases as functions of chemical potentials represented by the bulk composition and temperature.^115^ GB phase-like transitions^114,131^ can drastically alter the functional properties by changing the interfacial physical properties directly or altering microstructural evolution to change the materials’ properties subsequently. For example, GB resistance can be reduced via destabilizing the detrimental GB defect phase (e.g., “drying” silicate-based IGFs in oxygen-ion conductors^116,120,121^) or promoting grain growth to reduce the number of insulating GBs.

For solid electrolytes, GB phase-like transitions can drastically alter the ion conduction by causing interfacial structural transitions (e.g., inducing interfacial disordering) and/or changing the space charges in the abutting crystals by altering the charge accumulation at the interfacial core.^116^ Thus, an opportunity emerges to use GB phase-like transitions to tailor solid electrolytes.^116^

As an example, we discuss recent studies^132–134^ on improving the ionic conductivity of compositionally complex perovskite oxides via utilizing a GB disordering transition. In this case, the lithium ionic conductivity of a new class of compositionally complex perovskite oxides can be improved via a GB transition that can not only promote grain growth to reduce total GB resistance but also improve GB-specific conductivity directly (Figure 5b). Specifically, the ionic conductivity was improved by 60% in (Li_0.375_Sr_0.4375_)(Ta_0.375_Nb_0.375_Zr_0.125_Hf_0.125_)O_3-δ_ (LSTNZH), compared to the (Li_0.375_Sr_0.4375_)(Ta_0.75_Zr_0.25_)O_3-δ_ (LSTZ) baseline through enhancing the grain growth (Figure 5c). The temperature-dependent grain growth was examined to investigate the origin of exaggerated grain growth in Nb-containing LSTNZH. Notably, increasing temperature induces significant GB segregation of Nb (Figure 5d), in contrast to the classical GB segregation models that suggest temperature-induced desorption. Instead, it suggests the occurrence of premelting-like GB disordering, coupled with and enhanced by the GB segregation of the Nb_2_O_5_ (the binary oxide with the lowest melting temperature, 1512℃, in comparison with 1871℃ for Ta_2_O_5_ and >2700℃ for ZrO_2_ and HfO_2_). It also explains the observed abnormal grain growth, which reduces the total GB resistance by reducing the number of GBs. Furthermore, the specific GB ionic conductivity of LSTNZH can be further improved via quenching to preserve the (presumably more disordered) Nb-segregated GBs (Figure 5e). The combination of enhanced grain growth and improved specific GB conductivity resulted in GB-enabled conductivity improvements in quenched LSTNZH, achieving ~270% of the conductivity of the LSTZ baseline.

### GB transitions induced by applied electric fields

In addition to varying temperature or chemical potential, GB transitions can also be induced by applying electric fields via electrochemical coupling (i.e., chemo-electrical coupling resulting from an applied electric field).^135,136^ In 2021, Nie et al.^135^ reported that local reduction due to electrochemical coupling in Bi_2_O_3_-doped ZnO can induce a GB disorder-to-order transition. This GB transition stems from the Bi_2_O_3_-segregation-induced formation of amorphous-like IGFs (disordered GBs) and subsequent interfacial ordering upon cathode-side Bi reduction (noting that the reduction also occurred at one of the monocrystal-polycrystal interfaces in a polycrystal-monocrystal-polycrystal specimen due to the blocking of oxygen ion transport by the ZnO monocrystal, while the liquid-like GBs in Bi_2_O_3_-doped ZnO are ion-conducting), and it can cause abnormal and exaggerated grain growth near the cathode.^135,137^ We note that Bi_2_O_3_-doped ZnO is a prototypical varistor system with important energy applications, in which GBs and associated space charges play a critical role in governing the nonlinear current–voltage (I–V) characteristics (electron transport across GBs). Here, the observed GB transitions offer a new mechanism to tune GB structures and electronic properties, in addition to controlling microstructural evolution.

**Figure 6:**
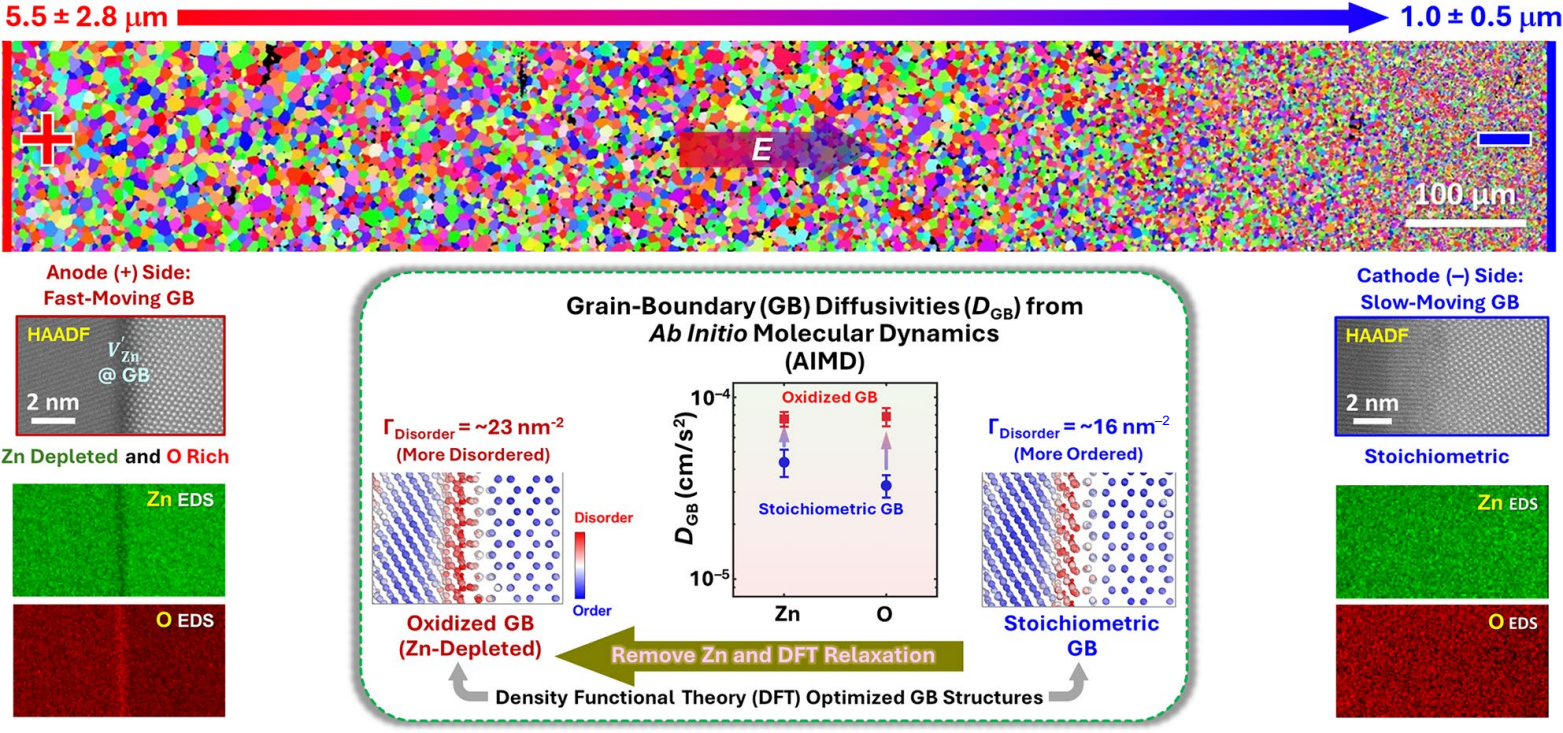
An example of a grain boundary (GB) transition induced by an applied electric field.^136^ In this specific example, an applied electric field induces a GB transition via electrochemical coupling (i.e., chemo-electrical coupling that creates extreme local oxidation/reduction conditions at the anode/cathode side), which is utilized to create graded microstructures. Specifically, an applied electric field induces a GB oxidation transition at the anode side in ZnO. Aberration-corrected scanning transmission electron microscopy (AC STEM) in conjunction with density functional theory (DFT) and *ab initio *molecular dynamics (AIMD), revealed cation-deficient, oxygen-rich GBs near the anode with enhanced GB diffusivities. This anode-side GB oxidation transition leads to the formation of cation-deficient (oxidized) GBs to gradually promote grain growth toward the anode. Such GB transitions may also alter GB ionic and electronic transport, as well as microstructural stability and reliability of electrochemical systems. HAADF, high-angle annular dark field. Adapted from Reference 136. © 2024 CC‐BY license.

 Yan et al.^137^ further reported that a GB oxidation transition can be induced by an applied electric field near the anode in undoped ZnO. Utilizing this GB oxidation transition that enhances grain growth, continuously graded microstructures are created under applied electric fields (**Figure ****6**). These studies suggest a new opportunity to induce GB phase-like transitions with applied electric fields via electrochemical coupling (i.e., transport and redistribution of ions under an applied electric field/current) to alter the microstructural evolution of functional materials. Such GB transitions may also change interfacial functional properties (e.g., conductivity). In addition, because many energy and functional devices are operating under electric fields at room or high temperatures (e.g., fuel cells), such GB transitions can also take place to affect stability and device reliability (e.g., causing intergranular lithium dendrite growth in solid-state batteries).

## Conclusions

GBs have a complex and multifaceted role in defining the functional behavior of energy materials, with impact across photovoltaic, thermoelectric, and battery applications, as explored in this article. Rather than being passive interfaces, GBs actively influence charge, heat, and ion transport through their atomic structure, local chemistry, and associated defect states. Examples of correlative microscopy studies using SEM, EBSD, EBIC, and APT highlight the potential to discover these influences at GBs, essential to achieving specific improved device properties, such as reduced recombination in solar-cell absorbers. The transport phenomena at GBs, including mass, thermal, electrical, and ionic transport mechanisms, are major contributors to specific GB properties, making them a key focus of this review. Three types of device materials serve as the basis for analysis, electronic transport mechanisms and the barrier height of GBs are analyzed in the context of solar-cell absorber materials, mass and ionic transport mostly in terms of battery materials, and thermal transport using thermoelectric materials. The formation of distinct interfacial phases and the variability of potential barriers, in conjunction with the direct connection to GB character, highlight the possibility of tailoring GB properties for specific applications. This may be achieved by modulating their chemical composition, structure, and carrier concentration.

## Supplementary Information

Below is the link to the electronic supplementary material.

Supplementary file1 (DOCX 2228 KB)
