# Supplementary material for: Structure and composition of grain boundaries and their impact on functional properties of energy materials
Source: MRS Bull. 2026 Feb 25;51(2):189–201. doi: 10.1557/s43577-025-01038-y (PMC12957157; doi:10.1557/s43577-025-01038-y)
Supplement: Supplementary file 1 — Supplementary file1 (DOCX 2228 KB) [file 43577_2025_1038_MOESM1_ESM.docx]

**Structure and composition of grain boundaries and their impact on functional properties of energy materials – Supplementary information**

Oana Cojocaru-Mirédin^1,^*, Elisa Wade^1^, Yuan Yu^2^, Jian Luo^3,4^*

^1^Department of Sustainable Systems Engineering (INATECH), University of Freiburg, Emmy-Noether-Straße 2, 79110 Freiburg, Germany

^2^ Institute of Physics (IA), RWTH Aachen University, 52056 Aachen, Germany

^3^AiisoYufeng Li Family Department of Chemical and Nano Engineering, University of California San Diego, La Jolla, CA 92093, USA

^4^Program in Materials Science and Engineering, University of California San Diego, La Jolla, CA 92093, USA

Corresponding authors*: [oana.cojocaru-miredin@inatech.uni-freiburg.de](mailto:oana.cojocaru-miredin@inatech.uni-freiburg.de) ; [jluo@alum.mit.edu](mailto:jluo@alum.mit.edu)

**Figure S1** shows a selection of atomic structures of several Σ9 GBs, both symmetric and asymmetric, in multicrystalline Si^1–3^. The structures were investigated using HAADF- and BF-STEM. The intermediate region between the grains is notably larger than in the previous Σ3 GBs, best visible in
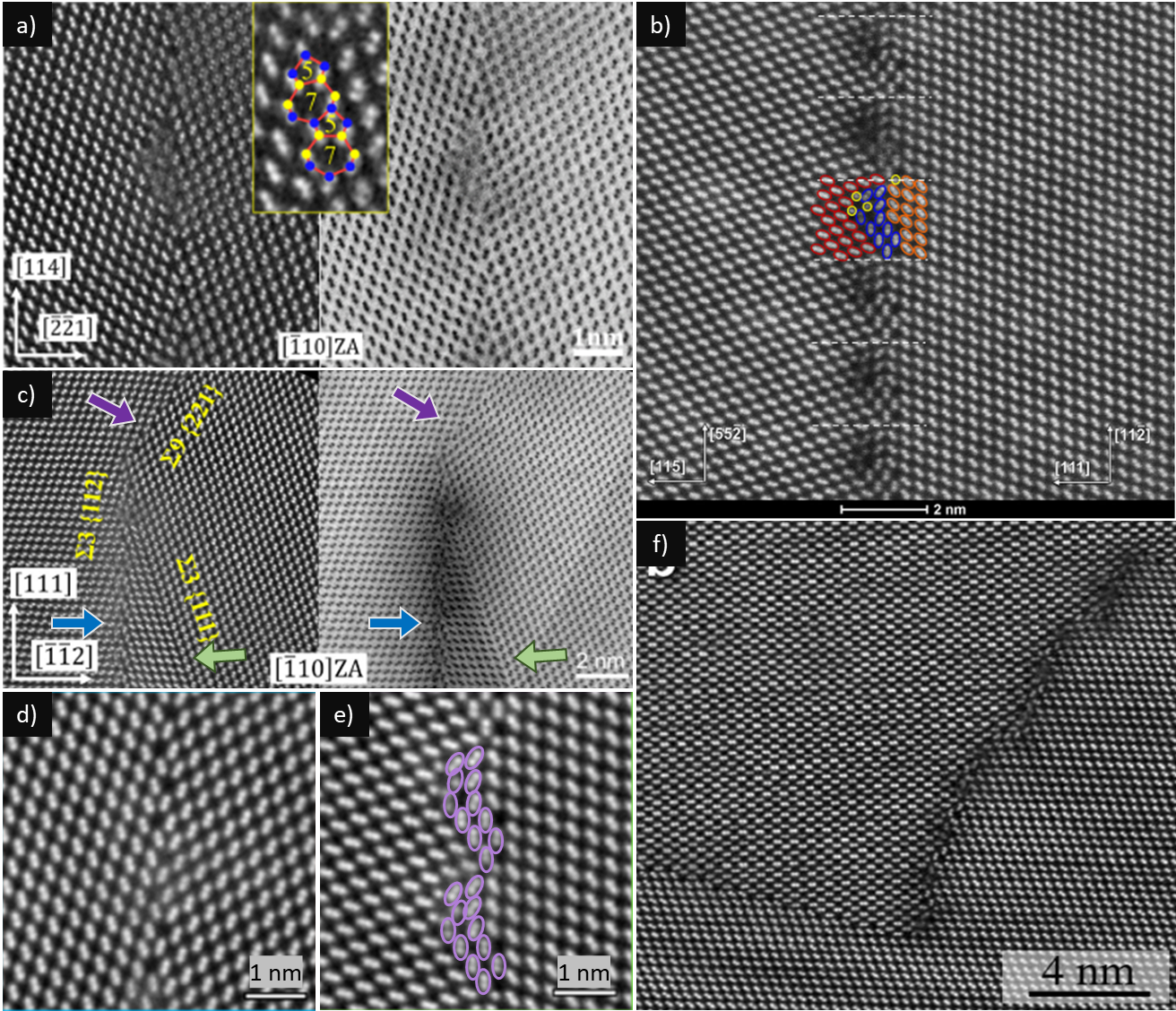
Figure S1b, e and f, where entire sections of atoms cannot be attributed to a specific grain. As demonstrated for Σ3 GBs, the intermediate atoms repeat periodically, best emphasized in Figure S1b, where Stoffers^2^ highlighted the periodic sequence in an asymmetric Σ9(111|115) GB segment, and Figure S1e, where the intermediate atoms are marked in purple. Figure S1 also includes more symmetric Σ9 segments, where the intermediate region is not as large, yet still of a similar periodicity.

**Figure S1:** Atomic structures of several Σ9 GBs in silicon. Some intermediate atoms not clearly attributed to either grain are highlighted in (b) and (e). (a) High-resolution HAADF-STEM (left) and BF-STEM (right) images of a Σ9 {221} GB in multicrystalline Si^1^. (b) High-resolution HAADF-STEM image of the asymmetric Σ9(111|115) GB segment in multicrystalline Si^2^. (c) HAADF-STEM (left side) and BF-STEM (right side) image of a GB triple junction of two Σ3 and one Σ9 GBs in multicrystalline Si^1^. (d-e) High-resolution HAADF images of symmetric Σ9 (221), and asymmetric (111)|(115) GBs, respectively, in multicrystalline Si.^3^ (f) HAADF-STEM-image of Σ9 interface consisting of {122} and incoherent {114} facets^2^. Panels (b) and (f) are adapted from ref ^2^ with permission. Panel (a) and (c) License status to be confirmed. Panels (d,e) are reproduced with permission from ACS, License No. 6098071124638.

1. M. G. Tsoutsouva, P. E. Vullum, K. Adamczyk, M. Di Sabatino, G. Stokkan, *J Appl Phys*. **127**, 125109 (2020).

2. A. Stoffers, thesis, RWTH Aachen (2017).

3. B. Haas, T. M. Boland, C. Elsässer, A. K. Singh, K. March, J. Barthel, C. T. Koch, P. Rez, *Nano Lett*. **23**, 5975–5980 (2023).
